# WNT and NOTCH signaling in human trophoblast development and differentiation

**DOI:** 10.1007/s00018-022-04285-3

**Published:** 2022-05-13

**Authors:** Bianca Dietrich, Sandra Haider, Gudrun Meinhardt, Jürgen Pollheimer, Martin Knöfler

**Affiliations:** 1 Placental Development Group, Department of Obstetrics and Gynaecology, Reproductive Biology Unit, Medical University of Vienna, Währinger Gürtel 18–20, 5Q, 1090 Vienna, Austria; 2 Maternal-Fetal Immunology Group, Department of Obstetrics and Gynaecology, Reproductive Biology Unit, Medical University of Vienna, Währinger Gürtel 18–20, 5Q, 1090 Vienna, Austria

**Keywords:** Placental development, Trophoblast stem cells, Trophoblast differentiation, WNT signaling, NOTCH signaling

## Abstract

Correct development of the human placenta and its differentiated epithelial cells, syncytial trophoblasts (STBs) and extravillous trophoblasts (EVTs), is crucial for a successful pregnancy outcome. STBs develop by cell fusion of mononuclear cytotrophoblasts (CTBs) in placental floating villi, whereas migratory EVTs originate from specialized villi anchoring to the maternal decidua. Defects in trophoblast differentiation have been associated with severe pregnancy disorders such as early-onset preeclampsia and fetal growth restriction. However, the evolutionary pathways underlying normal and adverse placentation are poorly understood. Herein, we discuss Wingless (WNT) and NOTCH signaling, two pathways that play pivotal roles in human placenta and trophoblast development. Whereas WNT is necessary for expansion of trophoblast progenitors and stem cells, NOTCH1 is required for proliferation and survival of EVT precursors. Differentiation of the latter is orchestrated by a switch in NOTCH receptor expression as well as by changes in WNT ligands and their downstream effectors.

## Introduction

The human placenta, a versatile organ, undergoes dynamic structural changes throughout pregnancy [1, 2]. It develops from stem cells of the extraembryonic trophectoderm (TE), the first epithelial cell layer that originates during human development surrounding the inner cell mass [3, 4]. Upon contact with the maternal endometrium, TE stem cells establish the primitive syncytium (PS) by lateral cell fusion. The PS erodes the uterine stroma, glands and vessels thereby allowing successful implantation of the blastocyst. Subsequently, the TE layer gives rise to primary villi consisting of stacks of mononuclear cytotrophoblasts (CTBs) breaching the PS [3]. CTBs then contact the maternal decidua underneath, expand laterally and merge thereby building the trophoblastic shell encircling the embryo [5]. At this stage, the first differentiated cell type of the maturing placenta, the extravillous trophoblast (EVT), develops. EVTs expand from the shell, migrate into the maternal spiral arteries and clog these vessels thereby preventing early oxygen delivery to the conceptus [6]. Defects in plugging and evolution of the shell could provoke premature blood flow and, as a consequence, oxidative stress of the placenta which might be a reason for early pregnancy losses [7–9]. During the first 3 weeks of gestation placental villi are then transformed into secondary and tertiary villi upon sequential colonization with mesenchymal stromal cells and vessels [10]. The placenta further develops by extensive proliferation and branching morphogenesis and continuously builds its differentiated cell types, syncytiotrophoblasts (STBs) and EVTs from stem and progenitor cells that are still poorly defined [11–13].

Both differentiated trophoblast subtypes, STB and EVT, play multiple roles during pregnancy [1]. Placental villi of early gestation develop a bi-layered epithelium consisting of villous CTBs (vCTBs) and STBs. Multinuclear STBs originate from underlying vCTB progenitors after asymmetrical cell division and fusion with the pre-existing syncytium and represent the transport interface between maternal and fetal circulation [13]. STBs secrete hormones such as human chorionic gonadotrophin into the maternal blood stream thereby adapting the endocrine system and metabolism of the mother [14, 15]. During the first 10 weeks of gestation STBs transport nutrients secreted by decidual glands to the developing embryo [16]. This process, termed histiotrophic nutrition, occurs in the absence of oxygen, thereby avoiding damage of the early placental villi by free oxygen radicals. After the 10th to 12th week of pregnancy, trophoblast plugs are dissolved, maternal blood enters the intervillous space and reaches the placental epithelium [17, 18]. Consequently, the placenta switches from histiotrophic to haemotrophic nutrition since subsequent growth of the embryo requires effective transport of oxygen and nutrients by the placental STBs. To increase the transport capacity, the STB layer gets thinner toward the end of pregnancy and the underlying placental vessels closely contact the epithelium thereby diminishing the distance for transport between the maternal and fetal blood stream [19].

Remodeling of the uterine spiral arteries, mediated by EVTs, is fundamental for establishing the blood flow between the mother and the placenta [20, 21]. While EVTs develop from the trophoblastic shell in the early phases of placentation, they originate from specialized villi anchoring to the maternal decidua later on. At contact sites with the decidua, proliferative cell columns (CCs) are formed consisting of progenitors of the EVT lineage [22]. In the distal region of the CC differentiated EVTs, herein termed placental EVTs (pEVTs), develop that quit proliferation and enter an endocycle provoking polyploidization [23]. Subsequently, EVTs detach from the CC, migrate into the decidual stroma as interstitial EVTs (iEVTs) and communicate with different uterine cell types [24]. They also invade the maternal spiral arteries of the decidua up to the first third of the myometrium [25]. In deeper regions of the uterus iEVTs form multinuclear giant cells which represent the end stage of the EVT differentiation process. Genome size differs between giant cells of decidua and myometrium suggesting that the extent of polyploidization could eventually determine their localization [26]. In the arterial vessels EVTs adopt a vascular adhesion phenotype and replace the maternal endothelial cells thereby lowering vasoconstriction [27, 28]. While endovascular EVTs (eEVTs) remodel the arteries from inside, iEVTs approach them from outside. Decidual macrophages (dMs) and uterine natural killer (uNK) cells initiate the transformation process and recruit iEVTs which then provoke apoptosis and degradation of the vessel wall [29, 30]. As a consequence the diameter of the spiral arteries increases thereby establishing low-pressure blood flow to the placenta. The latter provokes optimal perfusion and is thought to prevent oxidative stress, mechanical damage of villi, and a potential adverse pregnancy outcome. Indeed, defects in the remodeling process and impaired EVT invasion have been observed in different gestational disorders including preeclampsia, fetal growth restriction (FGR), pre-term labor and stillbirth [31–34]. Likely due to the turbulent blood reaching the intervillous space, branching of placental villi is diminished whereas placental apoptosis and STB turnover are elevated in preeclampsia and FGR [35].

In addition to their key roles in spiral artery remodeling, EVTs fulfill diverse tasks during placentation. During the early phases of pregnancy iEVTs breach the uterine glands, fostering histiotrophic nutrition, and secrete hormones and enzymes such as diamine oxidase (DAO) into the maternal circulation [36, 37]. Like factors expressed by STBs, EVT-secreted proteins could adjust the maternal metabolism to the needs of pregnancy. Moreover, iEVTs invade venous vessels and lymphatics of the decidua, the latter promoting fluid drainage and/or local immune cell trafficking [38, 39]. Less EVTs have been noticed in these vessels of patients with recurrent pregnancy loss, suggesting that interactions with different cells types of the maternal vasculature are crucial for successful placentation [39].

Fetal abnormalities and underlying maternal diseases, for example hypertension, are considered as causes of preeclampsia and FGR [40]. However, their severe forms such as early-onset preeclampsia could be largely associated with defects in placentation [41]. Unfavorable immunological interactions between specific paternal human leukocyte antigen (HLA)-C alleles and killer cell immunoglobulin-like (KIR) receptors, expressed on iEVTs and uNK cells, respectively, could impair the migratory capacity of EVTs [42]. Since the decidua controls EVT invasion in a paracrine fashion, defects in decidualization process could also contribute to incomplete spiral artery remodeling in preeclampsia and FGR [43, 44]. However, EVT differentiation might be directly affected in these diseases [35]. Indeed, accumulation of immature iEVTs at the basal plate and failures in integrin switching, that normally occurs when these cells deeply migrate into the decidua, has been observed in preeclamptic tissues [45, 46]. Moreover CTBs, isolated from women with this disease, also show molecular defects since key markers of EVTs such as HLA-G and MMP-9 were insufficiently expressed during spontaneous in vitro differentiation [47].

Despite the fact that placental development has profound effects on the success of pregnancy, its different evolutionary stages remain largely elusive. Normal placental tissues can only be obtained from legal pregnancy terminations between the 6th and 12th week and at the end of gestation upon delivery of the baby. Therefore, many phases of placental development remain a black box. Although histological examinations of human hysterectomy specimens and of animals with comparative placentation, such as primates, gave morphological insights into the early stages of human placental development [48, 49], its molecular regulation remains largely unknown. Likewise, a plethora of questions have only been partly resolved such as identification of placental trophoblast stem cells (TSCs) in situ, regulatory pathways controlling self-renewal and cell fate specification as well as key mechanisms governing formation of differentiated EVT subtypes.

For decades, primary human CTB progenitors have been isolated from first and term placental tissues and cultivated in vitro [50]. Whereas trophoblast cells of term placentae were utilized for studying regulation of STB formation and hormone expression, CTBs of early placentae were predominantly analyzed for their invasive capacity in the presence of numerous growth factors and cytokines produced by trophoblasts and/or the decidua [51–53]. However, the rapid loss of their proliferative capacity in culture and the inability to control spontaneous differentiation into either STB or EVT has hampered a deeper understanding of placental developmental processes. Yet in that regard, recent progress has been made by establishing self-renewing human TSCs and 3-dimensional (3D) trophoblast organoids (TB-ORGs) using pure CTB preparations obtained from early pregnancies [54–56]. In these models, conditions for progenitor proliferation and controlled EVT differentiation were delineated [54, 55].

As for other tissues and organs crucial developmental pathways such as transforming growth factor β (TGF-β), epidermal growth factor (EGF) and HIPPO signaling play pivotal roles in human placentation and trophoblast development. For example, the transcriptional co-activator of HIPPO signaling, Yes-associated protein (YAP), was recently shown to promote CTB expansion in cooperation with a key regulator of trophoblast development, TEA domain 4 (TEAD4), while it repressed STB formation [57, 58].

Whereas crucial signaling pathways have been discussed elsewhere [52, 53, 59], the present review specifically focusses on the role of the developmental signaling pathways Wingless (WNT) and NOTCH in the TE, in post-implantation CTBs as well as in differentiated trophoblast subtypes. It represents an update of previous reviews on WNT and NOTCH in human placentation [60–62], including features of these pathways in the novel TSC and TB-ORG models. Regulation and signaling through these pathways are highly complex due to the presence of numerous ligands, receptors and inhibitors. Moreover, both canonical and non-canonical WNT and NOTCH pathways, involving different signaling components and downstream effectors, have been described [63, 64]. Whereas ongoing research has primarily focused on the role of canonical WNT and NOTCH in human placentation, their non-canonical effects have been poorly investigated. Hence, the present review primarily focusses on the key components of canonical WNT and NOTCH signaling and their autocrine and paracrine regulations. Furthermore, we describe their specific functions in human trophoblast proliferation and differentiation in relevant primary trophoblast models, including isolated first trimester trophoblast cell types, villous explant cultures, TSCs and TB-ORGs. Whereas activation of canonical WNT signaling plays a crucial role in the expansion of TSCs and/or CTB progenitors, NOTCH1 signaling is required for proliferation and survival of precursors of the EVT lineage [22, 54]. Moreover, changes in WNT downstream effectors and in NOTCH receptor expression regulate differentiation of EVT progenitors into EVTs. Other potential models, such as trophoblastic cell lines, will not be discussed herein since they show aberrant expression patterns of WNT and NOTCH receptors, ligands and signaling components. They also differ considerably from primary CTB cultures with respect to HLA- and overall gene expression questioning their suitability as appropriate trophoblast systems [65, 66].

## Canonical WNT and NOTCH signaling: key components and downstream effectors

**Fig. 1:**
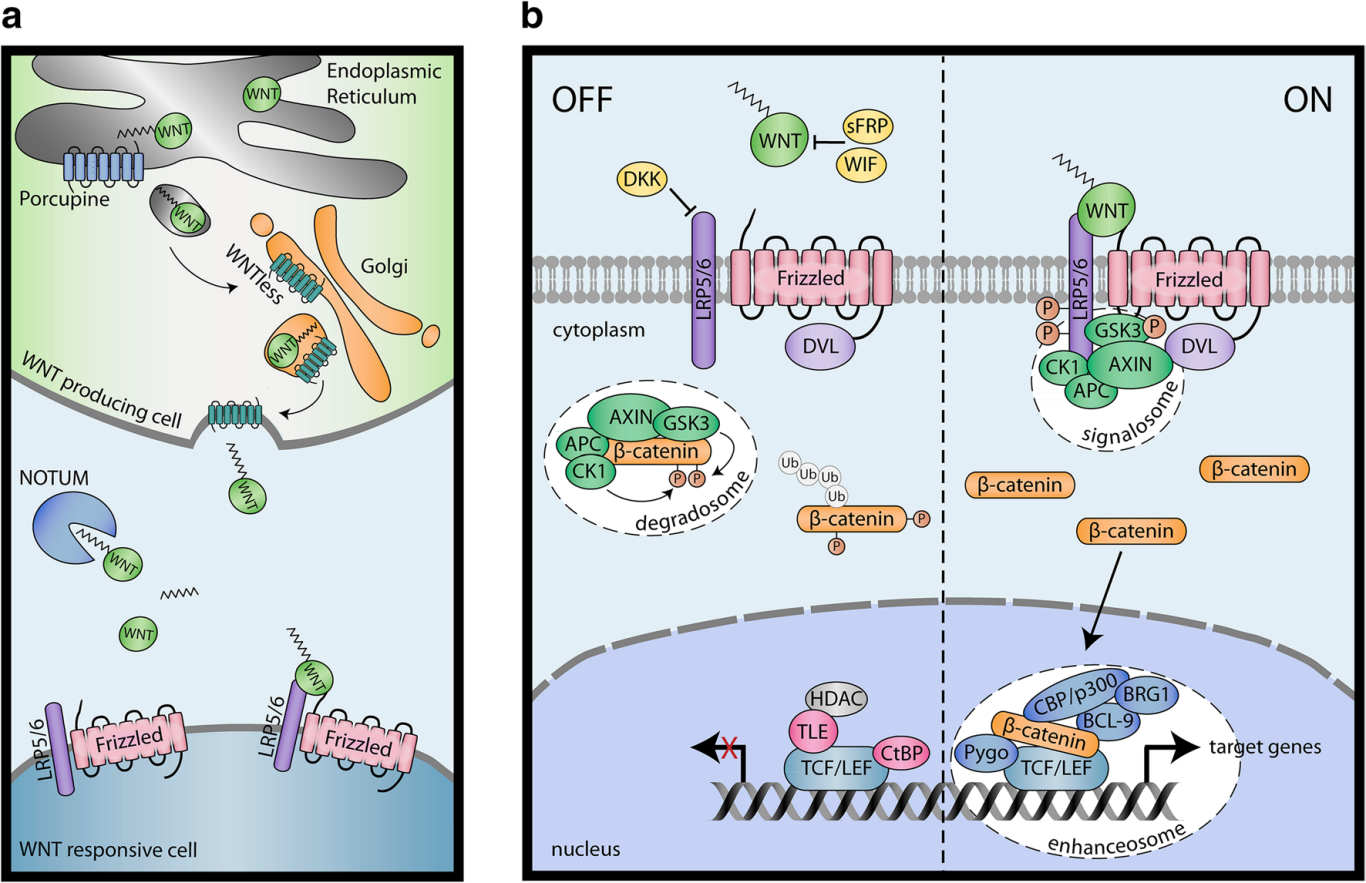
Key components of the canonical WNT signaling pathway. **a** Posttranslational modification and secretion of WNT ligands. Porcupine conjugates palmitoleate residues to WNTs, whereas NOTUM removes them. **b** ON-and OFF-state of canonical WNT signaling. In the absence of WNT ligands β-catenin is kept in the degradosome, while WNT activation provokes formation of a signalosome at the membrane, elevation of cytoplasmic β-catenin levels, its nuclear recruitment and formation of the β-catenin-dependent TCF/LEF enhanceosome. *APC* adenomatous polyposis coli, *BCL-9* B-cell lymphoma 9, *BRG1* Brahma-related gene-1, *CBP* CREB-binding protein, *CK1* casein kinase 1, *CtBP* C-terminal-binding protein, *DKK* Dickkopf, *DVL* Dishevelled, *HDAC* histone deacetylase, *GSK3* glycogen synthase kinase 3, *LEF* lymphoid enhancer-binding factor, *LRP5/6* low density lipoprotein receptor-related protein 5/6, *sFRP* secreted Frizzled-related protein, *Pygo* Pygopus, *TCF* T-cell factor, *TLE* transducin-like Enhancer of split, *WNT* Wingless, *WIF* WNT inhibitory factor

The WNT signaling pathway plays a pivotal role in stem cell maintenance, cell fate decisions and differentiation of tissues and organs [67]. WNT ligands comprise a family of 19 different secreted proteins that are posttranslationally modified by glycosylation and palmitoylation [68, 69]. Palmitate is added to WNTs by the O-acyltransferase Porcupine at the endoplasmic reticulum thereby conferring activity to the particular ligands (Fig. 1a). The modification allows for transport of WNTs by the cargo receptor WNTless into the extracellular space as well as for binding to the G-protein-coupled Frizzled (FZD) receptors [70]. Outside the cell the carboxylesterase NOTUM potentially removes the palmitoleate side chain from WNT thereby inhibiting its activity [68]. The FZDs represent a family of 10 different seven-helix-bundle receptors interacting with the different WNT ligands through their cysteine-rich domains [67, 71]. Several negative regulators of WNT signaling, blocking ligand–receptor interactions, have been identified, for example WNT inhibitory factor (WIF), Dickkopf (DKK) proteins and members of the secreted Frizzled-related protein (sFRP) family [72].

The critical key component of canonical WNT signaling is the protein β-catenin serving as a transcriptional co-activator in cells with activated WNT signaling (Fig. 1b). In the OFF-state cytoplasmic levels of β-catenin are low due to its degradation in a specific destruction complex, the degradosome. In this complex AXIN interacts with the tumor suppressor protein adenomatous polyposis coli (APC) providing a scaffold for the binding of β-catenin. Next, Ser/Thr residues in the N-terminus of β-catenin are sequentially phosphorylated by kinases casein kinase 1α (CK1α) and glycogen synthase kinase 3 (GSK3) thereby provoking conjugation of the β-TrCP E3 ubiquitin ligase, ubiquitination and degradation in the proteasome [71, 73]. Notably, the cytoplasmic forms of the transcriptional co-activators of HIPPO signaling, YAP and transcriptional coactivator with PDZ-binding motif (TAZ), are also part of the destruction complex and are required for the recruitment of the E3 ubiquitin ligase [74]. In the ON-state WNT ligands interact with the heterodimeric WNT receptors consisting of FZD and low density lipoprotein receptor-related proteins 5 or 6 (LRP5/6). WNT binding activates phosphorylation of LRP5/6 by CK1γ and GSK3 and recruitment of the destruction complex to the membrane via interaction of AXIN with LRP5/6. The multifunctional protein Dishevelled (DVL) binds to the cytoplasmic tail of FZD and facilitates binding of AXIN to LRP5/6 [75]. As a consequence, a WNT signalosome can be formed sequestering components of the destruction complex thereby preventing degradation of β-catenin [76]. The latter accumulates in the cytoplasm and translocates into the nucleus where it interacts with members of the lymphoid enhancer-binding factor (LEF)/T-cell factor (TCF) family [77]. In the absence of WNTs these transcription factors silence target genes by interacting with different repressors such as C-terminal-binding protein (CtBP) and members of the Groucho/transducin-like Enhancer of split (TLE) family, the latter recruiting histone deacetylases (HDACs) into the complex [78]. In WNT-stimulated cells β-catenin acts as a transcriptional co-activator binding the chromatin modifiers CREB-binding protein (CBP)/p300 and Brahma-related gene-1 (BRG1) as well as additional co-activators/scaffold proteins, e.g. B-cell lymphoma 9 (BCL9)/legless and Pygopus (PYGO) [77]. As a consequence WNT-activated LEF/TCF promotes expression of genes that control diverse biological processes during development such as cell cycle progression, migration and differentiation.

**Fig. 2:**
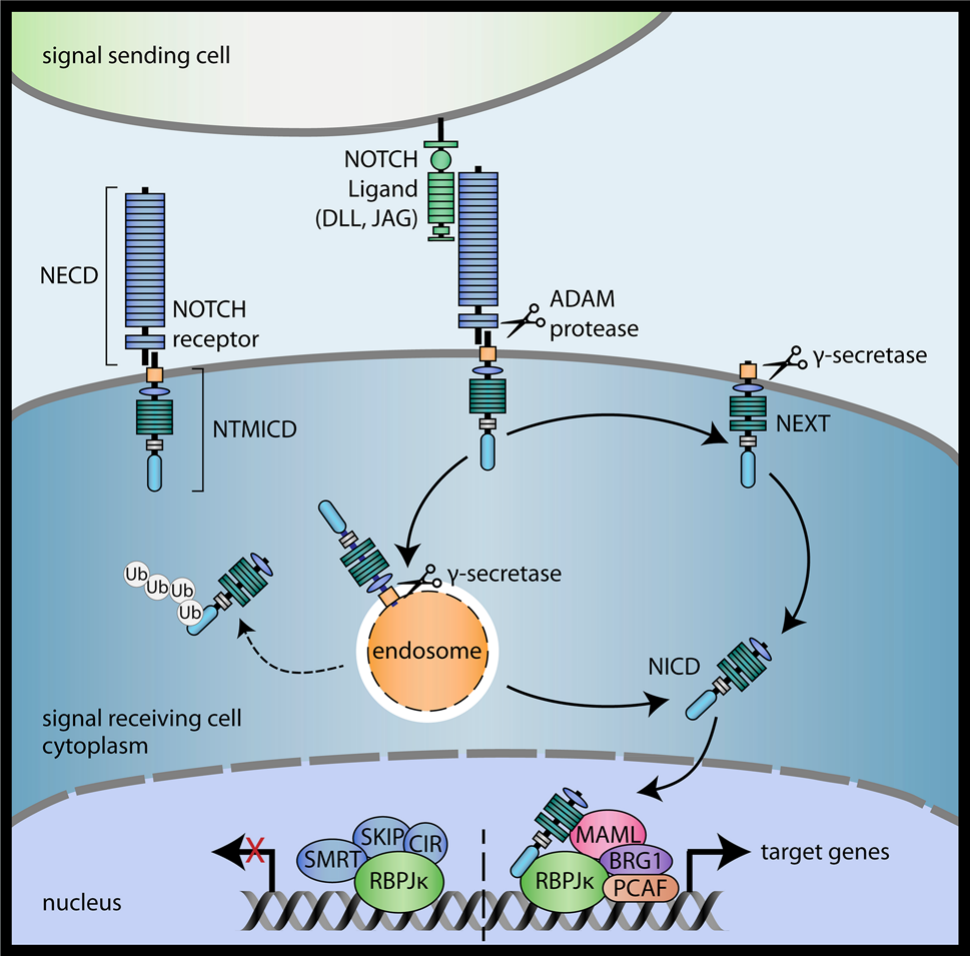
Schematic depiction of the canonical NOTCH signaling pathway. Interaction of membrane-bound NOTCH ligand with the NOTCH extracellular domain (NECD) of the dimeric NOTCH receptor provokes two sequential cleavage steps in the NOTCH transmembrane and intracellular domain (NTMICD) performed by ADAM proteins (NEXT) and γ-secretase (NICD). As a consequence, NOTCH intracellular domain (NICD) is released from membranes and translocates into the nucleus. NICD binds and converts the transcriptional repressor recombination signal binding protein for immunoglobulin kappa J (RBPJκ) into an activator. *ADAM* a disintegrin and metalloproteinase, *BRG1* Brahma-related gene-1, *CIR* CBF-interacting co-repressor, *MAML* Mastermind-like, *NEXT* NOTCH extracellular truncation, *PCAF* CBP-associated factor, *SKIP* Ski-interacting protein, *SMRT* silencing mediator of retinoic acid and thyroid hormone receptor

NOTCH signaling represents another developmental pathway that is crucial for tissue homeostasis and stem cell maintenance [79]. Differently to WNT, activation of NOTCH signaling requires direct cell–cell contact via its membrane-bound ligands and receptors [80]. The signal-sending cell expresses Serrate-like ligands Jagged1 and 2 (JAG1 and 2) or Delta-like ligands (DLL1, 3 and 4) that interact with the extracellular domain of NOTCH receptors (NOTCH1-4) via their EGF repeats (Fig. 2). NOTCH receptors are heterodimers consisting of the ligand-binding NOTCH extracellular domain (NECD) and the non-covalently linked NOTCH transmembrane and intracellular domain (NTMICD). Upon activation membrane-located ADAM (a disintegrin and metalloproteinase) proteins, such as TNFα converting enzyme, cleave NTMICD thereby generating the NOTCH extracellular truncation (NEXT). A further proteolytic cleavage step in the transmembrane domain by the γ-secretase complex generates the NOTCH intracellular domain (NICD) [81]. The particular protease could act at the cell surface or in the acidic milieu of endosomes upon intracellular trafficking of NEXT [79]. The latter is controlled by a negative regulator of NOTCH signaling, the multifunctional NUMB protein, which can target NOTCH for ubiquitination and proteasomal degradation [82]. After cleavage NICD is released from the membrane and migrates into the nucleus where it provides co-activator function upon binding to the transcription factor recombination signal binding protein for immunoglobulin kappa J (RBPJκ) via its RAM (RBPJκ-associated module) domain [80]. The RBPJκ-NICD complex then recruits Mastermind-like (MAML) and other co-activators, e.g. p300, BRG1 and the histone acetylase p300/CBP-associated factor (PCAF), replacing transcriptional repressors such as silencing mediator of retinoic acid and thyroid hormone receptor (SMRT), Ski-interacting protein (SKIP) or CBF-interacting co-repressor (CIR) [83, 84]. Thereby RBPJκ is converted into a transcriptional activator inducing expression of cell cycle proteins, survival genes, and regulators of differentiation. Most importantly, canonical NOTCH signaling provokes expression of members of the Hairy/Enhancer of Split (HES) and Hairy/Enhancer of Split-related with YRPW motif (HEY) families. HES and HEY proteins are transcriptional repressors that interact with Groucho/TLE proteins and downregulate expression of genes promoting cell fate determination and differentiation [85, 86]. Like the WNT pathway, NOTCH signaling is highly complex due to numerous non-canonical effects of NICD and its crosstalk to most other pathways controlling developmental processes. In that regard, diverse cross-regulatory interactions between WNT and NOTCH have been detected including phosphorylation of NICD by GSK-3 and regulation of active β-catenin by membrane-bound NOTCH [87–89].

## Expression of WNT and NOTCH and their roles in the human TE

During human embryogenesis TE development is initiated in outer cells of the morula stage. The cells acquire an apical-basal cell polarity by the activation of atypical protein kinase C controlling expression and nuclear localization of the Hippo co-activator YAP and the TE/trophoblast-specific transcription factor GATA binding protein 3 (GATA3) [90]. At the blastocyst stage enrichment of several WNT genes as well as activation of the pathway was observed in the TE compared to the inner cell mass (ICM), suggesting that WNT signaling could be involved in TE evolution and/or function [91, 92]. For example, transcripts of *WNT2B*, *WNT3*, *WNT4*, *WNT5A*, *WNT6*, *WNT11*, *FZD1*, *FZD9* and *DKK3* were found to be overrepresented in the TE compared to the inner cell mass (ICM) [91, 92]. In contrast, more recent single-cell RNA sequencing (RNAseq) data suggested a higher expression of *WNT3A*, *WNT5B* and *WNT7B* in the TE [93], whereas others detected enrichment of *WNT6*, *WNT7A*, *WNT7B* and *WNT16* [94]. Co-expression of *WNT7B* with the TE marker keratin 18 (*KRT18*) has also been detected in cluster analyses of blastocyst-derived single cells [95]. This cluster-specific expression pattern was already present at a low level in morula cells with a strong upregulation at the blastocyst stage. Collectively, the data suggest a possible role of WNT7B in human TE development and/or differentiation. However, functional evidence is lacking so far.

Studies in mice suggested that WNT-β-catenin is dispensable for blastocyst formation, while it is required for implantation [96, 97]. Accordingly, co-culture of human decidual cells with trophoblasts or their supernatants, mimicking implantation, affected expression of WNT signaling components [98, 99]. However like in mouse, activation of the canonical WNT pathway may not play a role in human blastocyst development since β-catenin is absent from nuclei of the TE [100]. In contrast, others suggested enhancement of the pathway in the TE of cows and humans relative to the ICM [101]. Proteins of the WNT pathway were also found to be enriched in human TE compared to the murine TE [102].

Yet, few studies have investigated the role of WNT and β-catenin in human pre-implantation TE development. In vitro degradation of β-catenin in human blastocysts at day 3 affected TE-specific expression of *KRT18* and of the key regulator of murine TE development caudal-related homeobox 2 (*CDX2*), whereas GSK-3 inhibition, activating canonical WNT signaling, diminished *GATA3* expression at day 6 [100]. Recent investigations suggested that WNT inhibition promotes a naïve human pluripotent stem cell (hPSC) state in combination with activated Activin- and Leukemia inhibitory factor (LIF) signaling as well as with blockage of several other pathways such as mitogen-activated protein kinase (MAPK) signaling [103, 104]. The naïve hPSCs represent many aspects of the pre-implantation epiblast and can be differentiated into self-renewing TSCs upon activation of canonical WNT signaling [105, 106]. TSCs obtained by this protocol resemble human blastocyst-derived TSCs [55], acquiring properties of the post-implantation TE. Alternative to MEK/ERK blockage, inactivation of γ-secretase or ablation of RBPJκ also allowed generating naïve hPSCs suggesting an inhibitory role for NOTCH in early human development [103]. Accordingly, blockade of NOTCH promoted growth of undifferentiated human embryonic stem cells, but also fostered TE/trophoblast marker expression upon BMP4-induced trophoblast differentiation [107]. Human TE cells express *NOTCH1*, *2*, *3* and *DLL4* as well as elevated transcript levels of *DLL3* and *JAG1* compared to ICM [91–93]. However, expression of NOTCH receptors and ligands might suggest a role in human implantation rather than in blastocyst formation. Indeed, a variety of studies indicated that NOTCH-ligand interactions play a crucial role in decidualization and implantation as discussed elsewhere [60, 108]. The role of NOTCH in murine TE development differs from human, since Notch1 ICD triggered TE formation in outer blastomeres and provoked Cdx2 expression in cooperation with Tead4-Yap1 transcriptional complexes [109].

## Canonical WNT signaling controls TSC/CTB progenitor expansion

Like other human epithelial stem cells that require WNT and EGF signaling for their proliferation, such as progenitors of the gut or the skin [110, 111], genes of these pathways were found to be overrepresented in placental CTBs [55]. This suggested that activation of WNT and EGF signaling could be necessary for long-term CTB expansion. Indeed self-renewing CTBs, growing in two dimensions (2D), could be established from human pre-implantation blastocysts and first trimester CTB preparations upon treatment with EGF, CHIR99021, a chemical GSK-3 inhibitor, A8301, a TGF-β signaling inhibitor, valproic acid (HDAC-inhibitor) and Y27632, blocking Rho-associated protein kinase (ROCK) [55]. While activation of canonical WNT signaling with CHIR99021 was indispensable for CTB proliferation, Y27632 was required for cell attachment. This contrasts the situation in mouse, where long-term expansion of murine TSCs required activation of fibroblast growth factor 4 (FGF4)-mediated signaling [112]. Amongst other specific criteria, such as hypo-methylation of the E74-like factor 5 (*ELF5*) promoter and expression of the chromosome 19 microRNA cluster, human TSCs expressed cytokeratin 7 (KRT7), TEAD4, p63, *TFAP2C*/AP-γ and GATA3 which have been delineated as in situ markers of proliferative vCTBs and/or trophoblast identity [55, 57, 58, 113–116]. The cells could also be differentiated into STBs and EVTs confirming their TSC/CTB progenitor status. Notably, TSCs derived from blastocysts displayed similar mRNA expression profiles as primary CTBs and placenta-derived TSCs suggesting that they have features of post-implantation trophoblasts [55]. In the same year when human TSCs were published, self-renewing 3D TB-ORGs, derived from pure first trimester CTB preparations were established [54, 56]. Conditions, similar to those for 2D TSCs, were utilized in these studies, whereas recent data showed that treatment with EGF, CHIR99021 and A8301 were sufficient for TB-ORG expansion [57]. While differentiation of 2D TSCs into STBs had to be triggered with forskolin, elevating cAMP levels, 3D TB-ORGs underwent spontaneous STB formation toward the centre [54–56]. As 2D TSCs, TB-ORGs expressed critical markers of trophoblast identity and vCTB proliferation such as the aforementioned key regulators GATA3, TEAD4, p63 and AP-2γ and displayed a hypo-methylated *ELF5* promoter region. Noteworthy, growing TSCs in 3D could be favorable compared to cultivation in 2D, since the latter provoked artificial upregulation of HLA-A and HLA-B, two proteins that are absent from all human trophoblast subtypes in vivo [117].

**Fig. 3:**
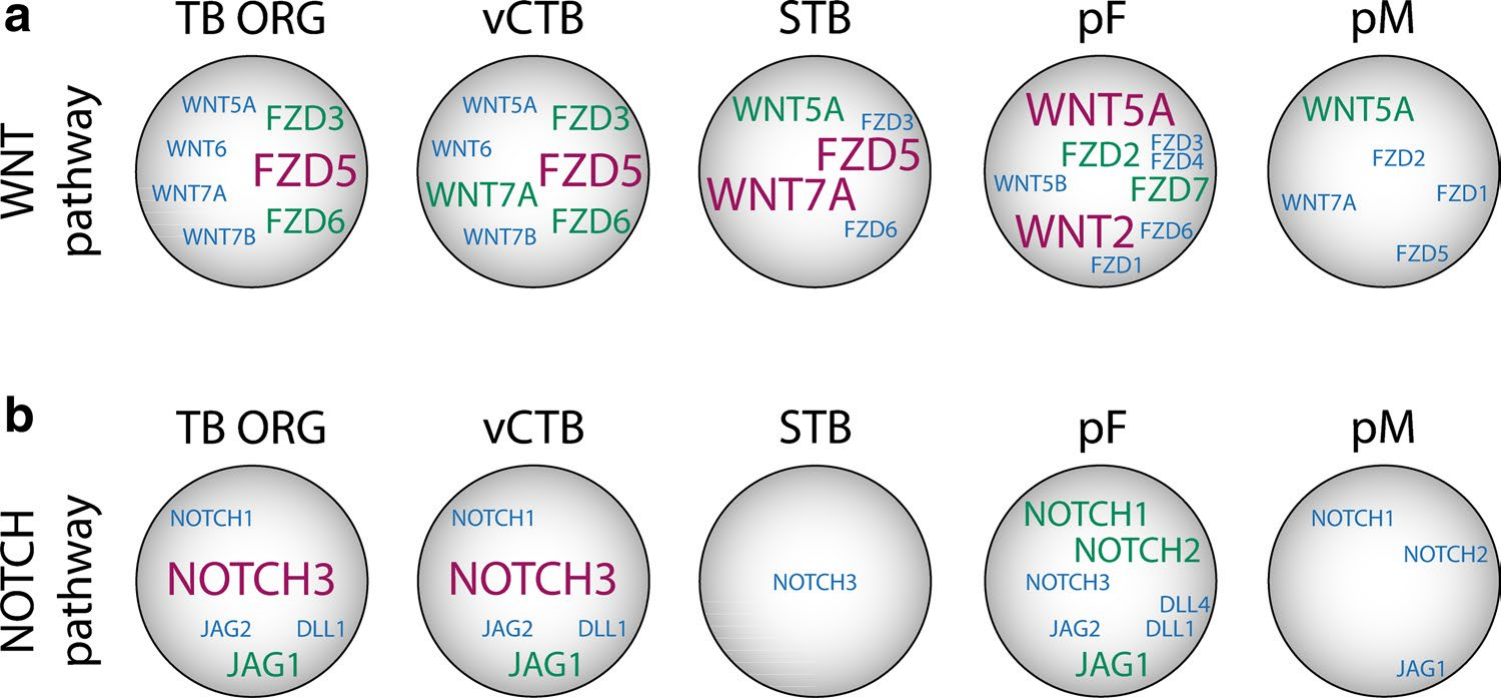
mRNA expression patterns of WNTs, FZDs, NOTCH ligands and receptors in the different cell types of first trimester placental villi. **a** Distribution of transcripts encoding WNTs and FZDs in trophoblast organoids (TB-ORG), villous cytotrophoblast (vCTB), syncytiotrophoblast (STB), placental fibroblast (pF) and placental macrophage (pM). **b** Expression of NOTCH ligands and receptors in the above mentioned cell types. Font sizes and colors indicate relative expression levels. Blue: low; green: medium; red: high

While the crucial role of canonical WNT signaling in the in vitro expansion of TSCs/CTB progenitors has been delineated, key players of the pathway in vivo remain largely elusive. Self-renewal of trophoblasts could be achieved by autocrine mechanisms and/or by paracrine effects of the underlying stroma. Whereas a study using semi-quantitative RT-PCR gave first insights into the expression pattern of WNTs and FZDs in the human placenta and its trophoblast subtypes [118], recent data obtained by bulk and single-cell RNAseq allow investigating WNT components in a quantitative manner. Herein, we present data on the expression levels of WNT ligands and FZD receptors that were taken from TB-ORGs and first trimester primary vCTBs, undergoing spontaneous cell fusion in vitro, from placental fibroblasts (pFs) of the villous core as well as from single-cell analyses of early placental tissues [54, 119]. These analyses suggest the expression of vCTB-restricted WNTs and FZDs, pF-enriched ligands and receptors and proteins that were common to both vCTB and pF (Fig. 3a). The pFs, underlying the CTB layer, represent a rich source for some of the WNTs suggesting that they could play a major role in TSC and CTB progenitor expansion. Eventually, placental macrophages (pMs)/Hofbauer cells, expressing WNT5A [120], could also exert paracrine effects on the villous epithelium. However, very few WNTs have been functionally analyzed in reliable trophoblast cell models such as villous explant cultures and primary CTBs. The first commercially available WNT protein with measurable activity in vitro was recombinant WNT3A. Although WNT3A is absent from the placenta it took over the function of endogenous WNT ligands by promoting cyclin D expression and growth of purified first trimester CTBs [121]. Inhibition of proliferation with recombinant DKK1 indicated that the canonical WNT pathway was involved [121]. Accordingly, nuclear β-catenin was detectable in a subset of CTBs of TB-ORGs and of vCTBs in situ [54]. The transcriptional co-activator β-catenin could activate WNT targets in CTBs by binding to TCF-1, TCF-3 or TCF-4, although the latter are predominantly expressed by EVTs [54, 121, 122]. TCF-4 might also play a role in trophoblast cell fusion. It was shown to bind to the promoter of the glial cells missing 1 (*GCM1*) gene, the key regulatory transcription factor in trophoblast syncytialization [123], and siRNA-mediated gene silencing of TCF-4 impaired choriocarcinoma cell fusion [124].

**Fig. 4:**
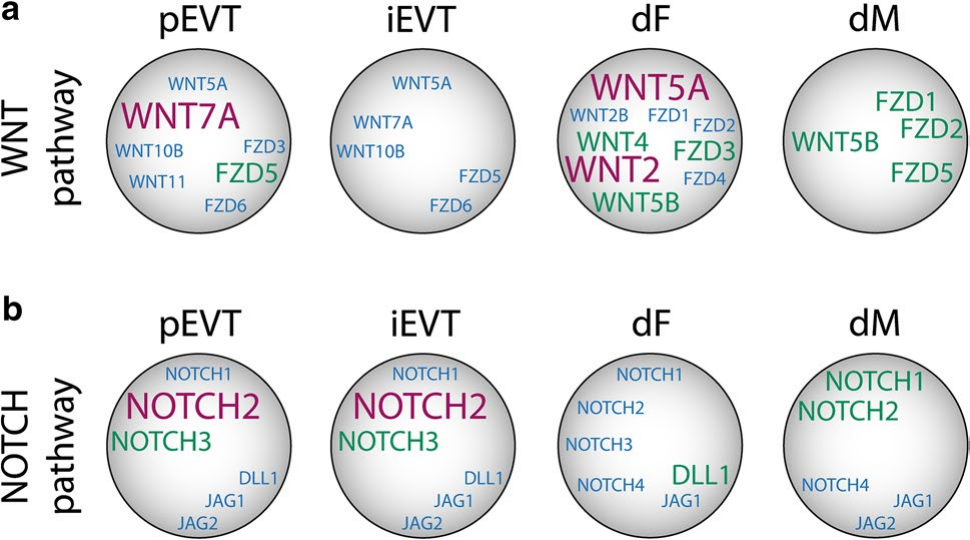
mRNA expression patterns of WNTs, FZDs, NOTCH ligands and receptors in the different cell types of first trimester anchoring villus and decidua. **a** Distribution of transcripts encoding WNTs and FZDs in placental EVT (pEVT), decidual interstitial EVT (iEVT), decidual fibroblast (dF) and decidual macrophage (dM). **b** Expression of NOTCH ligands and receptors in the above mentioned cell types. Font sizes and colors indicate relative expression levels. Blue: low; green: medium; red: high

Besides *WNT7A*, *WNT5A* seems to be the most widely distributed WNT among the different human placental cell types (Fig. 3a). While it is also present in vCTBs and EVTs, WNT5A protein is primarily expressed and secreted by pFs and decidual fibroblasts (dFs), suggesting a paracrine role in trophoblast growth (Figs. 3a, 4a) [120]. Indeed, WNT5A was shown to promote survival and proliferation of primary vCTBs and CC progenitors in villous explant cultures involving non-canonical activation of MAPK signaling [120]. Similarly, *WNT2*, expressed by pFs and dFs, could eventually trigger expansion of EVT/CTB progenitors and TSCs (Figs. 3a, 4a). In this context, discrimination of the latter remains enigmatic. While TSCs clearly show bipotential characteristics, developing into both differentiated trophoblast subtypes [55], the features of CTB progenitors remain uncertain. Like TSCs, CTBs, isolated from first trimester placenta, were shown to differentiate into STBs and EVTs suggesting that they might represent a homogenous bi-potential progenitor population [125]. However, classical CTB preparations represent a mixture of both vCTBs and EVT progenitors of the proximal CC. Indeed, recent evidence suggested that sequential trypsinization of early placental villi allows isolating pure vCTB and EVT precursors that develop into STB and EVT, respectively [22]. Accordingly, EVT progenitors purified from the CC cannot form STBs [126], suggesting that TSCs, precursors of STBs and progenitors of the EVT lineage are distinct cell types. The only marker so far that might discern TSCs and vCTB progenitors in the expanding villous epithelium is TCF-1. The particular WNT-downstream effector, which operates in different human stem cell niches, is expressed in a subset of first trimester vCTBs in situ suggesting that it could eventually mark the TSC population [54].

## Canonical NOTCH1 signaling controls EVT progenitor development.

Similar to WNTs and FZDs, NOTCH signaling components are differentially expressed between the distinct trophoblast subtypes of the placental villus. RNAseq data derived from previous investigations [54, 119] suggest that among the different NOTCH ligands and receptors *NOTCH3* and *JAG1* are the most abundant transcripts in first trimester vCTBs and self-renewing TB-ORGs, whereas *JAG2* and *DLL1* are expressed at low levels (Fig. 3b). The corresponding proteins have also been detected in first trimester vCTBs in situ, whereas vCTBs at a later stage of pregnancy lack JAG2 [127–129]. NOTCH4 could be another protein which is dynamically regulated since its expression increased in CTB progenitors during gestation [127, 128]. However, the specific role of NOTCH3 and the different NOTCH ligands in vCTB/TSC expansion remains unknown and awaits further investigations.

Another protein that rapidly declines during the first trimester of pregnancy is NOTCH1. Among trophoblasts NOTCH1 protein is exclusively expressed by EVT progenitors of the proximal CC, although its mRNA is widely distributed between the different placental and decidual cell types including EVTs, pFs, pMs, dFs and dMs [22, 54, 119] (Figs. 3b, 4b). NOTCH1 has been identified as the first gene controlling CC development [22]. Rapid downregulation of NOTCH1 protein in pEVTs of the distal CC might be explained by the presence of *NUMB* [119], fostering endocytosis, and the EVT-specific expression of ubiquitin ligases that promote proteasomal degradation of the receptor in other cellular systems [130–132]. NOTCH1 co-localizes with integrin α2 (ITGA2) thereby marking the EVT progenitor niche of the anchoring villus [133]. The *NOTCH1*^+^/*ITGA2*^+^ cell population has also been recently detected in TB-ORGs using single-cell RNAseq [134]. Moreover, NOTCH1 ICD was detectable in EVT progenitors and promoted proliferation and survival of these cells upon overexpression while differentiation into pEVTs was inhibited [22]. NOTCH1 ICD also induced the EVT progenitor phenotype since it activated CC-specific expression of MYC and VE-cadherin in vCTB progenitors, whereas the vCTB-restricted transcription factors TEAD4 and p63 were suppressed [22]. The latter was downregulated by NOTCH1 ICD-dependent expression of interferon regulatory factor 6 (IRF6), a negative regulator of p63 stability [135]. NOTCH1 and ITGA2 proteins were recently detected in TB-ORGs prepared from primary CTBs suggesting that some organoids, containing EVT progenitors, could be maintained under self-renewing conditions [117]. Notably, TB-ORGs derived from TSCs showed elevated expression of NOTCH1 and NOTCH1 ICD suggesting that the cells share features with EVT progenitors [117]. Alternatively, upregulation of the receptor could improve survival of the TSCs in 3D.

## Canonical WNT signaling regulates migration and differentiation of EVTs

Considering their dynamic roles in balancing cell proliferation and differentiation, it might not be surprising that WNT and NOTCH also control EVT differentiation and function. Herein, we analyzed previously published bulk RNAseq data of HLA-G^+^EVTs/pEVTs, purified from first trimester placental tissues [136], as well as single-cell RNAseq data of early placenta and decidua [119]. In the latter, the cluster of iEVTs can be identified by the expression of pregnancy-associated plasma protein A2 (PAPPA2) and the histamine-degrading enzyme DAO. In situ PAPPA2 and DAO are largely absent form pEVTs and specifically expressed and secreted by decidual iEVTs [37, 137]. These analyses revealed that *WNT7A* and *FZD5* were the most abundant WNT components in pEVTs (Fig. 4a). Previous investigations suggested that formation of pEVTs was associated with the upregulation of TCF-3 and TCF-4 as well as with the disappearance of membrane-bound E-cadherin and β-catenin [121, 122]. The latter was recruited into the nucleus when pEVTs detached from the distal CC. Accordingly, WNT promoted invasion of first trimester CTBs through Matrigel-coated transwells which could be inhibited with DKK1 suggesting that the canonical WNT pathway is crucial for trophoblast motility [121]. Silencing of TCF-4 in differentiating CTBs, expressing PYGO2, not only diminished basal and WNT3A-stimulated migration, but also downregulated expression of the EVT markers ITGA1, ITGA5, SNAIL and NOTCH2 [122]. Additionally, WNT3A was shown to activate non-canonical AKT signaling in villous explant cultures promoting MMP2 expression, outgrowth and migration [138]. Hence, the combined data suggest that differentiation of CC progenitors into pEVTs could be controlled by autocrine upregulation of WNT7A and its potential downstream effectors TCF-3 and TCF-4. As part of the epithelial to mesenchymal transition (EMT)-like process that cells undergo during EVT lineage formation [139], TCF-3/4-β-catenin transcriptional complexes promote migration and invasion. Compared to pEVTs transcript levels of *WNT7A* and *FZD5* decreased in iEVTs suggesting that autocrine WNT signaling could diminish when pEVTs have invaded the decidua (Fig. 4a). However, iEVTs encounter dFs, expressing high levels of *WNT2*, *WNT4* and *WNT5B* (Fig. 4a), which could regulate trophoblast motility in a paracrine fashion, whereas WNT5A, another abundant WNT in dFs, did not affect migration [120]. Yet DKK1, a progesterone-controlled gene, is also strongly expressed by dFs [140, 141]. The inhibitor could impair EVT motility, providing a mechanism for limiting trophoblast invasion in deeper regions of the decidua. Along those lines, iEVTs produce large amounts of the WNT-inactivating enzyme *NOTUM*, as visible in the single-cell RNAseq data [119], providing another putative mechanism to downregulate WNT signaling in distal areas of the placental bed.

Overall, the role of WNT in the developing anchoring villus seems highly complex. While WNT activation is crucial for the expansion of TSCs and progenitors in 2D and 3D TB-ORGs, its inhibition is necessary for EVT lineage formation [54, 55]. Unfortunately, 2D TSCs do not represent an appropriate system to study the precise role of the WNT pathway at the different developmental stages of anchoring villus formation and differentiation. Yet, investigations in TB-ORGs, undergoing CC formation and EVT differentiation in a correct 3D orientation, allowed deciphering the pivotal roles of WNT in the early human placentation. Noteworthy, removal of CHIR99021 (WNT^−^ condition) provoked formation of proliferative cell columns expressing the EVT progenitor marker NOTCH1 [54]. The latter then differentiated into HLA-G^+^ pEVTs at the outer rim of TB-ORGs expressing EVT markers such as NOTCH2, proteoglycan 2 (PRG2) and erythroblastic oncogene B (ERBB2) [39, 142, 143]. Like in tumor cells, the latter is amplified at genomic level in pEVTs and suppresses apoptosis upon hetero-dimerization with ERBB3 and activation with decidua-derived neuregulin 1 [142, 144].

Whereas TCF-1 was the predominant WNT-dependent regulator in self-renewing TB-ORGs, NOTCH1^+^ EVT progenitors did not express LEF or any of the TCF transcription factors [54]. However like in vivo and in differentiating primary CTBs, TB-ORG-derived pEVTs strongly expressed TCF-4 suggesting that the canonical WNT pathway was reactivated when the precursors underwent EVT differentiation [54, 122]. Indeed blockage of autocrine WNT activation with inhibitor of WNT response 1 (IWR-1), 2 days after the removal of CHIR99021, provoked accumulation of NOTCH1^+^ EVT progenitors and inhibited EVT formation in TB-ORGs [54]. Therefore, WNT signaling has a dual role in the developing anchoring villus. While it is not required for evolution and expansion of the proximal CC, it is necessary for TSC/vCTB proliferation as well as for EVT differentiation and invasion.

## EVT subtype-specific activation of NOTCH signaling

Similar to the WNT downstream effectors, NOTCH receptors undergo dynamic changes during EVT differentiation. CTBs quit proliferation in the distal CC, downregulate NOTCH1 protein and develop into polyploid pEVTs expressing NOTCH2 [23, 143]. Like vCTBs, first trimester pEVTs additionally expressed NOTCH3, DLL1, JAG1 and JAG2 transcripts (Fig. 4b) and proteins [127]. A similar pattern of *NOTCH* receptors and ligands was observed in decidual iEVTs (Fig. 4b), as retrieved from the single-cell analyses [119]. DLL1 and JAG1 have also been detected in endothelial cells of first trimester decidual vessels [143]. Distribution of NOTCH ligands seems to change during pregnancy since DLL1 is only expressed by maternal cells at later stages of gestation, whereas JAG1 is mainly present in iEVTs approaching the spiral arteries [128]. *DLL1* also seems to be the most abundant NOTCH ligand expressed by dFs (Fig. 4b) suggesting that its interaction with NOTCH2 and/or NOTCH3 could regulate EVT differentiation and/or function. Interestingly, NOTCH2 ICD has been detected in eEVTs of decidual spiral arteries suggesting that NOTCH2 activation could play a role in endovascular invasion and vessel remodeling [143]. Indeed, conditional knock-out of Notch2 in mice, impaired invasion of glycogen cells and trophoblast giant cells into spiral arteries and diminished placental perfusion [128]. Along those lines, antibody-mediated inhibition or gene silencing of NOTCH2 was shown to increase the migratory capacity of primary CTBs [143]. However, NOTCH2 ICD could not be detected in pEVTs in situ. Since binding of NOTCH to its ligands also has a crucial role in cell adhesion, independently of canonical signaling [145], a role of the receptor in maintaining CC integrity was speculated.

However, much remains to be learned about the specific roles of the diverse NOTCH receptors and ligands in human placentation. With the exception of NOTCH2, none of the other receptors or ligands haven been tested in appropriate primary EVT models. Downstream of NOTCH the co-activating MAML proteins also show differential distribution across the anchoring villus. MAML1 and MAML3 as well as the prime transcription factor of canonical NOTCH signaling, RBPJκ, were present in the proximal CC and pEVTs in situ, whereas MAML2 was predominantly expressed by pEVTs [146]. Notably, gene silencing of RBPJκ increased proliferation of CTBs and EVT progenitors and promoted outgrowth in villous explant cultures suggesting a inhibitory role of the factor in trophoblast expansion [146]. While these data seem to contrast the role of NOTCH1-ICD-RBPJκ in EVT progenitor induction and maintenance, one has to mention that RBPJκ acts as a repressor in the absence of NICD, as described above (Fig. 2), and has multiple NOTCH-independent functions [147].

Although not investigated in the human placenta yet, the cross-talk between WNT and NOTCH could play a role in human trophoblast development and differentiation. For example, MAML proteins have been detected in TCF-β-catenin transcriptional complexes [148]. Hence, they could also act as co-activators of TCF-4-β-catenin in pEVTs. Moreover, active β-catenin can be negatively regulated by binding to membrane-bound NOTCH [87]. In this context, it is worth mentioning that the sharp downregulation of NOTCH1 in pEVTs coincides with nuclear recruitment of β-catenin [22, 122]. In summary, multiple routes of investigations can be chosen to further delineate the complex roles of WNT and NOTCH in human placentation.

## Conclusions

**Fig. 5:**
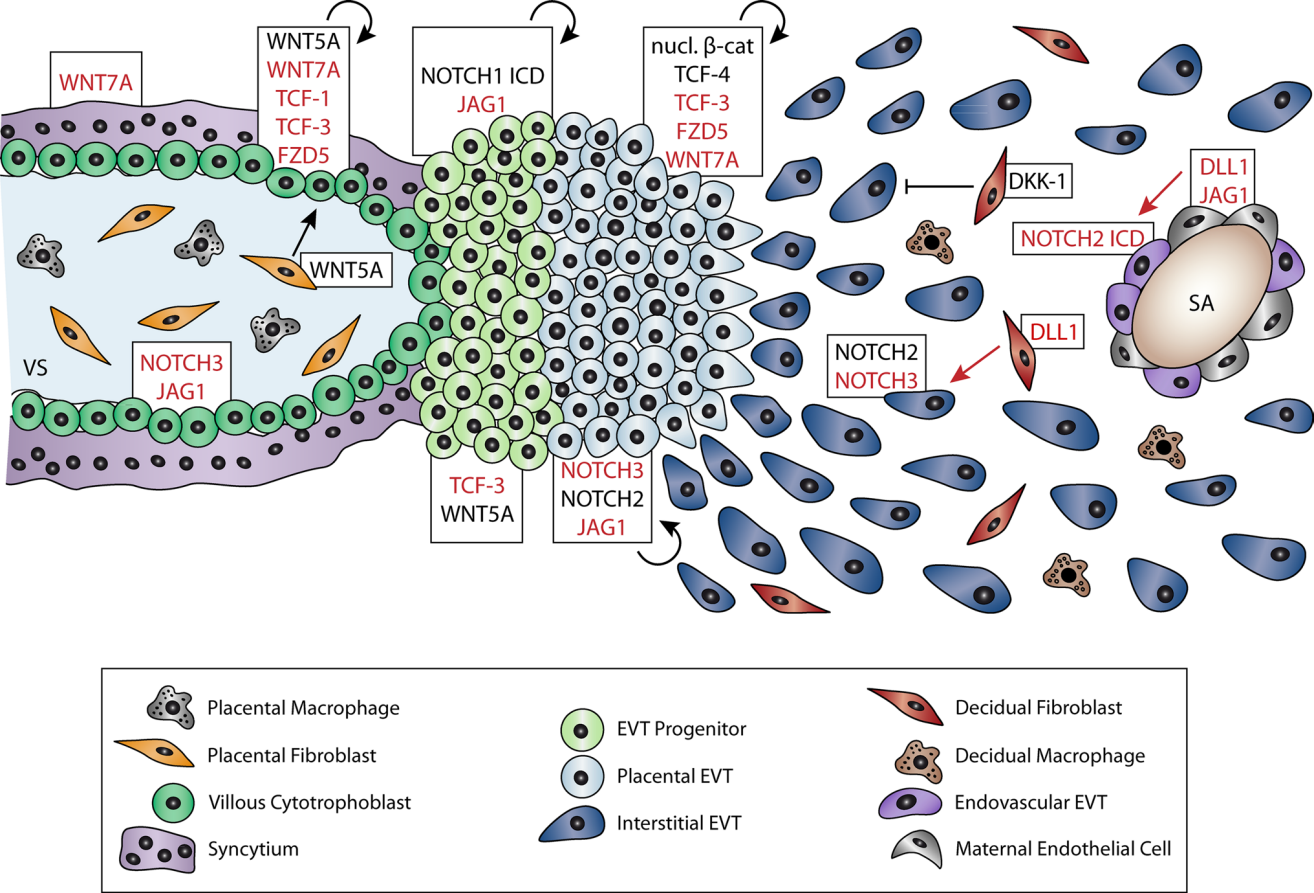
The role of WNT and NOTCH signaling in human trophoblast development and differentiation. Black arrows indicate autocrine (circular arrow) and paracrine (straight arrow) activation of progenitor expansion and differentiation, based on functional analyses in relevant primary trophoblast models. Red arrows depict possible interactions between cell types that still require functional evidence. WNT and NOTCH signaling components depicted in black indicate genes that have been experimentally tested in primary CTBs/EVTs, villous explant cultures, TSCs or TB-ORGs. Others, shown in red, illustrate receptors, ligands and downstream effectors that have only been analyzed at level of gene expression (protein and/or mRNA). *SA* spiral artery

NOTCH and WNT signaling play crucial roles in trophoblast development and differentiation of the human placenta. Whereas the tasks of these pathways in trophoblast cell fusion await further analyses, the integration of data obtained from different versatile primary models gave insights into their roles in trophoblast progenitor expansion and EVT differentiation (Fig. 5). In addition, bulk and single-cell RNAseq data of placental and decidual cells allows speculating on the role of WNTs, FZDs, NOTCH receptors and ligands in the different trophoblast subtypes [54, 119, 136]. Expansion and survival of vCTBs and TCSs could be mediated by WNT5A and WNT7A in a paracrine and/or autocrine manner, respectively. These WNTs could activate canonical signaling through FZD5 which is the predominant WNT receptor in placental trophoblasts. A role for FZD5 has also been delineated in mouse placentation where it controls GCM1 expression and branching morphogenesis [149]. TCF-3 and particularly TCF-1 could be the executing downstream factors in vCTBs and TCSs. The role of NOTCH3 and JAG1 in CTB proliferation and differentiation needs functional analyses. TCF-3 and WNT5A expressed in the proximal CC and by dFs, respectively, could play a role in EVT progenitor expansion through non-canonical mechanisms. Noteworthy, NOTCH1 is the only factor known so far that initiates development of EVT progenitors and promotes their growth and survival. Differentiation of EVT progenitors into pEVTs is associated with reactivation of canonical WNT signaling and a switch in the expression of TCF factors and NOTCH receptors. While TCF-4 promotes EVT differentiation and invasion in an autocrine manner, possibly through WNT7A-FZD5, NOTCH2 exerts inhibitory effects on EVT migration. WNT-dependent invasion might be restrained by decidual DKK1. Finally, canonical NOTCH2 signaling is activated in eEVTs suggesting a role in vessel remodeling. In conclusion, the role of WNT and NOTCH in trophoblast development and differentiation is slowly emerging. While WNT5A, TCF-4 and NOTCH1 have been investigated in detail [22, 120, 122], the role of other receptors and ligands have been hardly elucidated. Follow up studies should address their specific functions in CTB progenitor expansion and differentiation and also decipher their non-canonical roles in human trophoblast development.

## Data Availability

Not applicable.
